# Another Emergent Cause of Headache

**DOI:** 10.7759/cureus.2623

**Published:** 2018-05-14

**Authors:** Scott R Wambolt, Juan Garza

**Affiliations:** 1 Emergency Medicine, San Antonio Military Medical Center; 2 Emergency Medicine, UT Health Science Center San Antonio

**Keywords:** headache, chronic myeloid leukemia, leukostasis, retinal hemorrhage, hydroxyurea, accelerated phase

## Abstract

We present a case of a subacute headache related to leukostasis secondary to accelerated chronic myeloid leukemia (CML), which required white blood cell (WBC) reduction in the emergency department. A 28-year-old male presented to the emergency department with a chronic headache found to be secondary to leukostasis from accelerated CML with a white blood cell count of 801,000 and 9% blasts. He had bilateral retinal hemorrhage and a headache associated with elevated intracranial pressure. Hydroxyurea and allopurinol were initiated in the emergency department and the patient was eventually transitioned to a tyrosine kinase inhibitor as outpatient therapy. Headaches are a frequent cause of emergency department visits, and this case illustrates another possible etiology of headache requiring emergent intervention.

## Introduction

Headaches are common, though leukostasis secondary to malignancy is an extremely rare etiology. This report is regarding an emergency department presentation for a subacute headache as a result of leukostasis from chronic myeloid leukemia (CML). CML is a myeloproliferative disorder characterized by a translocation between chromosomes 9 and 22, creating the Philadelphia chromosome. The result is the fusion of the BCR-ABL gene, causing uninhibited tyrosine kinase activity and subsequent granulocyte proliferation. CML has three phases: chronic, accelerated, and blast crisis [1-2]. Leukostasis typically occurs in the blast crisis phase (greater than 20% blasts) with hyperleukocytosis (greater than 100,000 white blood cells (WBCs)) [3]. Between 5% and 30% of adult leukemia cases present with hyperleukocytosis. The mechanism of leukostasis is not fully elucidated [4]. The reasons that a blast crisis seems more likely to trigger leukostasis are two-fold. The first is that blast cells are larger than mature cells and, therefore, are more likely to decrease flow and increase viscosity. The other is that blast cells are believed to secrete cytokines that activate endothelial cells and promote blast cell adhesion to the endothelium [5]. Other complications of hyperleukocytosis include tumor lysis syndrome (TLS) and disseminated intravascular coagulation (DIC). This case details a presentation of accelerated phase CML that resulted in a headache, retinal hemorrhage, and findings of elevated intracranial pressure (ICP) due to hyperleukocytosis without a blast crisis.

## Case presentation

**Figure 1:**
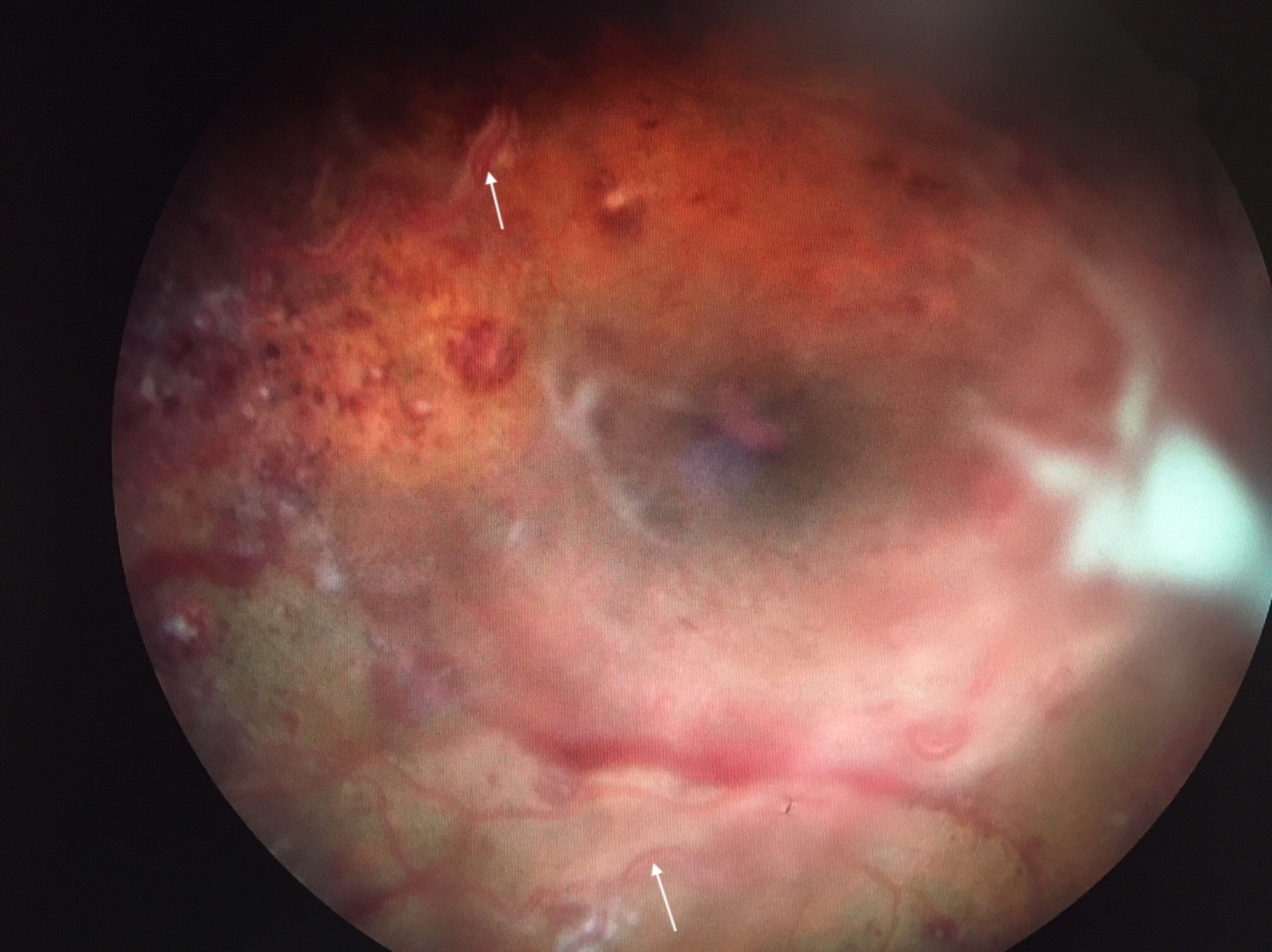
Image of the Right Fundus The right eye has a poor view, but the macula is visible centrally. Intraretinal hemorrhages are present throughout, with central collections of white blood cells forming Roth's spots. The optic nerve is difficult to visualize, given the overlying white blood cell collection in the vitreous cavity, anterior to the optic nerve. The lumen of the arterioles (arrows) has a white hue, secondary to the extremely high white blood cell count.

**Figure 2:**
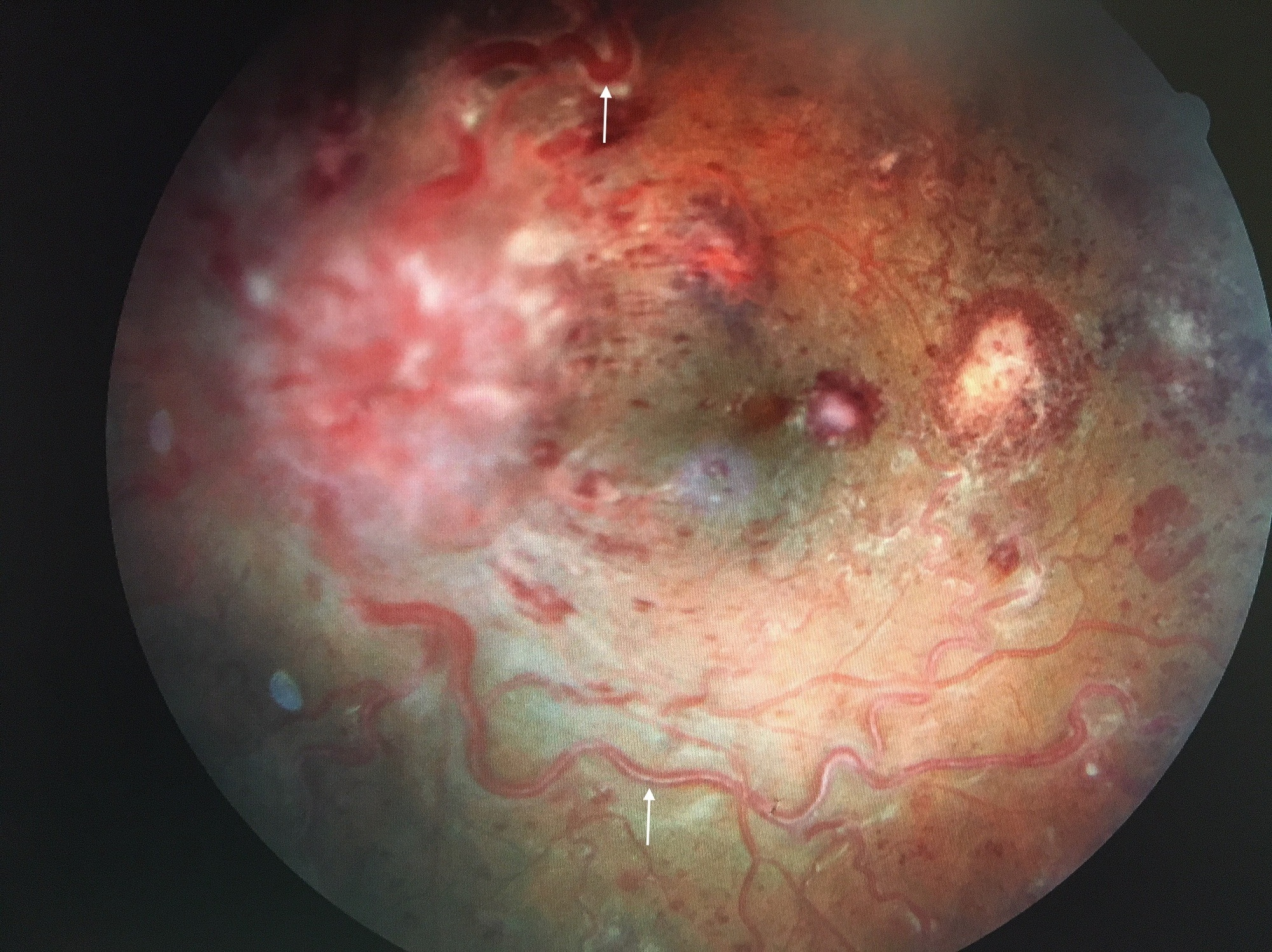
Image of the Left Fundus The left eye shows similar findings with Roth's spots and a whitening of the arterioles (arrows). The optic nerve demonstrates 4+ disc edema with anterior swelling, and is, thus, out of focus.

A 28-year-old Caucasian male with no known past medical history presented to the emergency department with a headache for six weeks. The pain was throbbing, changed locations, and was associated with mild nausea and intermittent generalized weakness, photophobia, and blurred vision. Vital signs and the physical examination were unremarkable at the time of presentation; a fundoscopic exam was not performed on initial evaluation. The patient attributed his headache to possible mold exposure in his apartment or recent smoking cessation. He did not initially have signs or endorse symptoms concerning for the life-threatening etiology of his headache, to include mass, intracerebral hemorrhage (ICH), or infection [6]. The initial differential diagnosis was broad, but the etiology appeared to be benign. The patient subsequently had improvement with metoclopramide and diphenhydramine. Laboratory studies and head computed tomography (CT) without contrast were ordered at triage. The reason they were ordered is unclear, as there were no clear red flags on presentation. The patient had a WBC count of 773,000 (801,000 on repeat laboratory draw) with a basophilic predominance (51%). Concern shifted to leukemia as the likely etiology of headache, with potentially a blast crisis causing leukostasis. Ophthalmology and hematology/oncology were consulted. On repeat history after laboratory studies, the patient endorsed multiple episodes of intermittent complete loss of vision lasting several seconds over the preceding few weeks, as well as recent night sweats and unintentional weight loss. Peripheral smear showed 9% blasts and had findings consistent with chronic CML, including basophilic predominance. Ophthalmologic examination demonstrated gross papilledema and retinal hemorrhage bilaterally, with a serous elevation of the right retina and turbid white cells below (Figures 1-2). The ophthalmologic findings were consistent with a head CT without contrast that was concerning for elevated ICP.

Leukocytapheresis, or white blood cell removal, was considered due to the degree of hyperleukocytosis. As the patient was not in an acute blast crisis, he was instead started on hydroxyurea and allopurinol in the emergency department, as per recommendations by hematology/oncology [7-8]. In addition, he was given aggressive intravenous fluids with the aim of hemodilution and decreasing blood viscosity. Allopurinol was started to prevent TLS on initiation of hydroxyurea [9]. The final diagnosis by the hematology team was accelerated phase CML, given the gross elevation of leukocytosis and concurrent symptoms, despite only having 9% blasts (13). Subsequently, BCR-ABL1positive CML was identified with a bone marrow biopsy. The patient received hydroxyurea until the WBC count fell below 50,000 and was later transitioned to outpatient dasatinib, a tyrosine-kinase inhibitor [10]. On discharge 17 days later, the WBC count was within normal limits.

## Discussion

Gross leukocytosis and subsequent leukostasis resulted in this patient’s presentation of a chronic headache with vision changes, night sweats, and weight loss. This is an uncommon headache etiology. This hematologic emergency required aggressive intravenous fluids, initiation of white blood cell reducing agents (hydroxyurea), and prophylaxis for tumor lysis syndrome. Marked leukocytosis (greater than 100,000) does not generally require emergent reduction unless the patient is symptomatic, denoting leukostasis rather than simply hyperleukocytosis. The clinical manifestations of leukostasis most commonly manifest within the central nervous and respiratory systems. Neurologic symptoms range from headaches, tinnitus, or dizziness, to blurry vision, confusion, and decreased mentation. Respiratory symptoms are nonspecific as well, including dyspnea, hypoxemia, tachypnea, and respiratory failure. It is also possible to have manifestations of ischemia from leukostasis, including myocardial infarction, limb ischemia, and bowel ischemia. Marked leukocytosis in CML is most commonly attributed to a blast crisis but can result from advanced chronic disease, as in this case. If the leukocytosis is due to a blast crisis, leukocytapheresis is another possible treatment option [5]

## Conclusions

Leukemia, and leukostasis, in particular, is a very uncommon etiology of headaches in the emergency department. This case highlights the importance of keeping the differential diagnosis broad, taking an adequate and detailed history, and pursuing a more thorough workup when no etiology for subacute or chronic headaches is identified. It also identifies hematologic malignancy as a potential cause of subacute headaches, one that may require emergent intervention. Overall, a thorough history of the present illness with a physical examination can help diagnose even the rarest of disorders.
